# Musculoskeletal manifestations in childhood-onset systemic lupus erythematosus: an in-depth exploration

**DOI:** 10.1186/s13052-024-01725-7

**Published:** 2024-08-16

**Authors:** Maynart Sukharomana, Siritida Vonginyoo, Nuntawan Piyaphanee, Sirirat Charuvanij

**Affiliations:** 1 Division of Rheumatology, Department of Pediatrics, Faculty of Medicine Siriraj Hospital, Mahidol University, 2 Wanglang Road, Bangkok Noi, Bangkok, 10700 Thailand; 2 Department of Pediatrics, Faculty of Medicine Siriraj Hospital, Mahidol University, Bangkok, Thailand; 3 Division of Nephrology, Department of Pediatrics, Faculty of Medicine Siriraj Hospital, Mahidol University, Bangkok, Thailand

**Keywords:** Arthritis, Joint pain, Musculoskeletal, Rheumatology, Systemic lupus erythematosus

## Abstract

**Background:**

Childhood-onset systemic lupus erythematosus (c-SLE) is a multifaceted autoimmune disorder predominantly affecting the musculoskeletal (MSK) system. This investigation delineated the spectrum and sequelae of MSK involvement in c-SLE patients.

**Methods:**

This retrospective analysis included SLE patients aged ≤ 18 years treated at a tertiary center between 2009 and 2019. Data were extracted from electronic health records.

**Results:**

The cohort comprised 321 SLE patients (mean age 13.2 ± 2.5 years, 91.3% female). MSK manifestations were observed in 134 (41.7%) individuals, with joint pain universally present, followed by joint swelling in 32.1% and morning stiffness in 9.7%. Arthritis was documented in 52 (38.8%) patients, whereas 82 (61.2%) had arthralgia. Symmetrical joint involvement was observed in 96 (71.7%) subjects. The knees, wrists, and fingers were most commonly affected, with incidences of 43.3%, 40.3%, and 33.6%, respectively. Neither erosive arthritis nor Jaccoud’s arthropathy was detected. MSK symptoms were significantly correlated with older age at diagnosis, the presence of non-scarring alopecia, neuropsychiatric manifestations, and elevated SLE disease activity index scores at diagnosis. Over a median follow-up of 53.6 months (IQR 26.1–84.6), five patients developed septic arthritis or osteomyelitis, and avascular necrosis was identified in 16 (4.9%) patients.

**Conclusions:**

Nearly half of c-SLE patients demonstrated MSK manifestations, chiefly characterized by symmetrical involvement of both large and small joints without evidence of erosive arthritis or Jaccoud’s arthropathy. Avascular necrosis is a critical concern and warrants close monitoring.

## Introduction

Systemic lupus erythematosus (SLE) is a multifaceted inflammatory autoimmune disorder of the connective tissue that is characterized by its propensity to affect multiple organs, especially the musculoskeletal (MSK) system. The prevalence of MSK manifestations in SLE patients is notably high, documented in up to 95% of cases, and these manifestations may emerge either at the initial diagnosis or during subsequent disease flares [1–4]. Joint pain, in particular, has emerged as a predominant symptom that significantly disrupts daily activities in patients with SLE [5]. The spectrum of lupus arthropathy encompasses various phenotypes [2], with patients experiencing joint pain devoid of overt inflammation, a condition referred to as arthralgia. Alternatively, some patients may develop arthritis, characterized by the involvement of both small and large joints in a typically symmetrical polyarthritis pattern [6, 7].

While the MSK symptoms in the majority of SLE patients are generally mild, manifesting as nondeforming nonerosive arthritis [2], a subset of patients may exhibit severe erosive joint inflammation, termed “Rhupus syndrome” [1, 6–8]. Chronic inflammation in SLE can also lead to tendon laxity and subsequent joint deformity, known as Jaccoud’s arthropathy [1, 7]. Beyond arthritis, inflammation may extend to adjacent soft tissues, inducing conditions such as periarthritis, tendinitis, tenosynovitis, myalgia, and myositis [1, 8]. The incidence of MSK-related complications in patients with SLE ranges between 4 and 12% [9, 10]. Additionally, the use of corticosteroids and immunosuppressive medications presents further risks, such as avascular necrosis, diminished bone mass, septic arthritis, and osteomyelitis [9, 11].

Research specifically dedicated to exploring MSK manifestations and their associated complications in childhood-onset SLE (c-SLE) patients is scarce, and findings may not align with those from adult cohorts [12]. Moreover, these data in c-SLE in the context of the Southeast Asian population are limited. Consequently, the objectives of this study were to explore the clinical manifestations of MSK involvement at initial presentation and the MSK complications throughout the disease course in c-SLE patients. Insights gleaned from our research are anticipated to encourage early recognition of MSK manifestations and reduce the incidence of MSK complications, ultimately advancing patient care and improving the quality of life for individuals with c-SLE.

## Methods

### Study design and setting

This retrospective cohort study was conducted at the Department of Pediatrics, Faculty of Medicine Siriraj Hospital, Mahidol University, in Bangkok, Thailand. Siriraj Hospital is recognized as the largest tertiary university-based referral center in the region. Data collection involved a thorough retrospective analysis of electronic medical records spanning from 2009 to 2019.

### Participants

The study cohort consisted of children and adolescents under 18 years of age who were diagnosed with SLE. These patients were classified according to either the 1997 American College of Rheumatology criteria [13] or the 2012 Systemic Lupus International Collaborating Clinics criteria [14]. The exclusion criterion was a diagnosis of mixed connective tissue disease or overlap syndromes.

### Data collection, variables, and outcome measurement

The collected baseline demographic and clinical information included sex, age at SLE onset and diagnosis, and initial SLE clinical manifestations, distinguishing between MSK and non-MSK manifestations. Initial laboratory assessments included a complete blood count, erythrocyte sedimentation rate, C-reactive protein levels, urinalysis, antinuclear antibody, anti-double-stranded deoxyribonucleic acid, anti-Smith antibody, antiphospholipid antibodies, direct antiglobulin test, and complement levels. The Systemic Lupus Erythematosus Disease Activity Index 2000 (SLEDAI-2K) [15] was used to evaluate disease activity.

In this study, arthritis was identified through objective evidence of joint swelling or the presence of at least two of the following criteria: restricted joint movement, joint tenderness, motion-induced pain, and warmth [16]. Arthralgia was characterized by joint pain without any objective signs of inflammation. The classification of joint involvement was as follows: monoarticular for a single joint (monoarthritis and monoarthralgia), oligoarticular for 2–4 joints (oligoarthritis and oligoarthralgia), and polyarticular for the involvement of five or more joints (polyarthritis and polyarthralgia).

MSK complications observed throughout disease progression were documented. Avascular necrosis was diagnosed through clinical assessment and verified by magnetic resonance imaging results. Bone mineral density (BMD) assessments were conducted using dual-energy X-ray absorptiometry. A BMD-Z score ≤ -2 standard deviations, adjusted to the normal reference values for Thai children [17] and height age for those with short stature, was defined as low bone mass.

### Ethical approval

This study was approved by the Siriraj Institutional Review Board (certificate of approval Si 660/2019) and was conducted in accordance with the Declaration of Helsinki. Due to the retrospective study design, the informed consent and assent were waived by the Siriraj Institutional Review Board.

### Sample size calculation

The sample size was derived using the findings from Watson et al. [11], who reported MSK manifestations in 82% of SLE patients. The formula used was *n* = Z^2^_α/2_p(1-p)/e^2^ (with a type I error [α] of 0.04, allowable error [e] of 0.05, Z_α/2_ = Z0.025 = 1.96, and p = 0.82). The required sample size was determined to be 354.

### Statistical analysis

IBM SPSS Statistics, version 28 (IBM Corp, Armonk, NY, USA), was utilized for the data analysis. We employed descriptive statistics to summarize the data. Categorical variables are expressed as frequencies and percentages, while continuous variables are presented as the means ± standard deviations for normally distributed data or as medians with interquartile ranges for non-normally distributed data. To compare differences between patients with and without MSK manifestations, we used the independent samples t-test for normally distributed continuous variables and the Mann–Whitney U test for those variables that were not normally distributed. Categorical variables were analyzed using the chi-square test and Fisher’s exact test. A *P* value less than 0.05 was considered to indicate statistical significance. Our reporting adheres to the Strengthening the Reporting of Observational Studies in Epidemiology guidelines [18].

## Results

This study included 321 SLE patients, with a female predominance in 293 (91.3%). The average age at diagnosis was 13.2 ± 2.5 years, with a median disease duration of 53.6 months (IQR 26.1–84.6). MSK manifestations were present in 134 (41.7%) patients at the initial diagnosis. Patients with MSK manifestations had significantly older age at diagnosis, longer duration from onset of SLE symptoms to SLE diagnosis, higher prevalence of non-scarring alopecia, neuropsychiatric involvement, and higher SLEDAI-2K (all *p* < 0.05). In contrast, those without MSK manifestations had higher prevalence of anemia, thrombocytopenia, renal involvement, hypocomplementemia, with lower mean hemoglobin levels and lower median platelet counts (all *p* < 0.05). Table 1 presents the baseline clinical characteristics of patients with childhood-onset SLE, comparing those with and without MSK manifestations.

**Table 1:** Baseline clinical characteristics of childhood-onset systemic lupus erythematosus patients: a comparative analysis of individuals with and without musculoskeletal manifestations (*N* = 321)

Parameters*	Overall (*N* = 321)	MSK manifestation (*n* = 134)	Non-MSK manifestation (*n* = 187)	*P*
Sex				0.498
Male	293 (91.3)	10 (7.5)	18 (9.6)	
Female	28 (8.7)	124 (92.5)	169 (90.4)	
Age at SLE diagnosis (year)	13.2 ± 2.5	13.7 ± 2.5	12.8 ± 2.3	**0.001**
Duration from onset to SLE diagnosis (month)	1.0 (0.8–1.9)	1.0 (0.9–2.4)	1.0 (0.7–1.3)	**0.012**
Duration of SLE disease (year)	4.4 (2.1–7.0)	4.6 (2.6–7.5)	4.0 (1.8–6.6)	**0.029**
SLEDAI-2K	11.3 ± 5.0	11.9 ± 5.3	10.7 ± 4.7	**0.031**
Mucocutaneous manifestation				
Acute cutaneous lupus	164 (51.1)	74 (55.2%)	90 (48.1%)	0.210
Chronic cutaneous lupus	66 (20.6)	31 (23.1%)	35 (18.7%)	0.334
Oral ulcer	132 (41.1)	59 (44.0%)	73 (39.0%)	0.370
Nonscarring alopecia	39 (12.1)	22 (16.4%)	17 (9.1%)	**0.048**
Serositis	52 (16.2)	17 (12.7%)	35 (18.7%)	0.148
Hematologic				
Anemia	85 (26.5)	24 (17.9)	61 (32.6)	**0.003**
Leukopenia	154 (48.0)	65 (48.5)	89 (47.6)	0.872
Thrombocytopenia	57 (17.8)	17 (12.7)	40 (21.4)	**0.044**
Renal	179 (55.8)	55 (41.0)	124 (66.3)	**< 0.001**
Neuropsychiatric	18 (5.6)	12 (9.0)	6 (3.2)	**0.027**
Complete blood count				
Hemoglobin (g/dL)	9.6 ± 2.0	9.9 ± 1.9	9.3 ± 2.1	**0.012**
WBC (cells/mm^3^)	5100 (3595, 8540)	5050 (3500, 7905.5)	5100 (3650, 9440)	0.480
ALC (cells/mm^3^)	1298 (882, 1880)	1309.50 (843, 1610.2)	1287 (900, 2048)	0.304
Platelet count (cells/mm^3^)	226 000 (137 000, 316 500)	260 000 (167 000, 321 250)	206 000 (108 000, 295 000)	**0.015**
ESR (mm/h)	59 (34, 82.5)	61 (40, 89)	56.5 (31, 80)	0.123
CRP (mg/L)	4.6 (1.7, 10.5)	6.5 (3.9, 16.9)	1.6 (1.0, 9.2)	0.123
Presence of anti-dsDNA	281 (87.5)	119 (88.8)	162 (86.6)	0.561
Presence of anti-Smith	44 (13.7)	20 (14.9)	24 (12.8)	0.591
Presence of APS	85 (26.5)	35 (26.1)	50 (26.7)	0.901
Presence of DAT	61 (19.0)	30 (22.4)	31 (16.6)	0.191
Hypocomplementemia	255 (79.4)	99 (73.9)	156 (83.4)	**0.037**

*ALC* absolute lymphocyte count, *anti-dsDNA* anti-double-stranded deoxyribonucleic acid, *APS* antiphospholipid antibody, *CRP* C-reactive protein, *DAT* direct antiglobulin test, *ESR* erythrocyte sedimentation rate, *SLE* Systemic lupus erythematosus, *SLEDAI-2K* Systemic Lupus Erythematosus Disease Activity Index 2000, *WBC* white blood cell

^*^Displayed as mean ± standard deviation, median (interquartile range), or n (%)

Table 2 provides an in-depth overview of MSK manifestations among patients with c-SLE. Joint pain emerged as the universal symptom, affecting 134 (100%) patients, followed by joint swelling in 43 (32.1%) patients and morning stiffness in 13 (9.7%) patients. Symmetrical joint involvement was observed in 96 (71.7%) patients, with a predominance of large joint afflictions in 88 (65.7%) patients. The knees, wrists, and fingers were the most frequently impacted joints, with involvement in 58 (43.3%), 54 (40.3%), and 45 (33.6%) patients, respectively. Myalgia and myositis were the least common MSK manifestations, present in 8 (6%) and 3 (2.2%) patients, respectively. No cases of erosive arthritis or Jaccoud’s arthropathy were identified.

**Table 2:** Spectrum of musculoskeletal manifestations, pattern of joint involvement, joint location, and muscle involvement in childhood-onset systemic lupus erythematosus patients (*N* = 134)

Musculoskeletal manifestations	n (%)
Joint pain	134 (100)
Arthralgia	82 (61.2)
Monoarthralgia	18 (13.4)
Oligoarthralgia	38 (28.4)
Polyarthralgia	26 (19.4)
Arthritis	52 (38.8)
Monoarthritis	5 (3.7)
Oligoarthritis	17 (12.7)
Polyarthritis	30 (22.4)
Joint swelling	43 (32.1)
Morning stiffness	13 (9.7)
Limping	1 (0.7)
Pattern of joint involvement	
Large joint	88 (65.7)
Small joint	46 (34.5)
Symmetrical	96 (71.7)
Asymmetrical	35 (26.1)
Axial	3 (2.2)
Location of peripheral joint involvement	
Knee	58 (43.3)
Wrist	54 (40.3)
Finger	45 (33.6)
Ankle	35 (26.1)
Elbow	28 (20.9)
Shoulder	10 (7.5)
Talus	4 (3.0)
Hip	3 (2.2)
Toe	3 (2.2)
Myalgia	8 (6.0)
Myositis	3 (2.2)

**Fig. 1:**
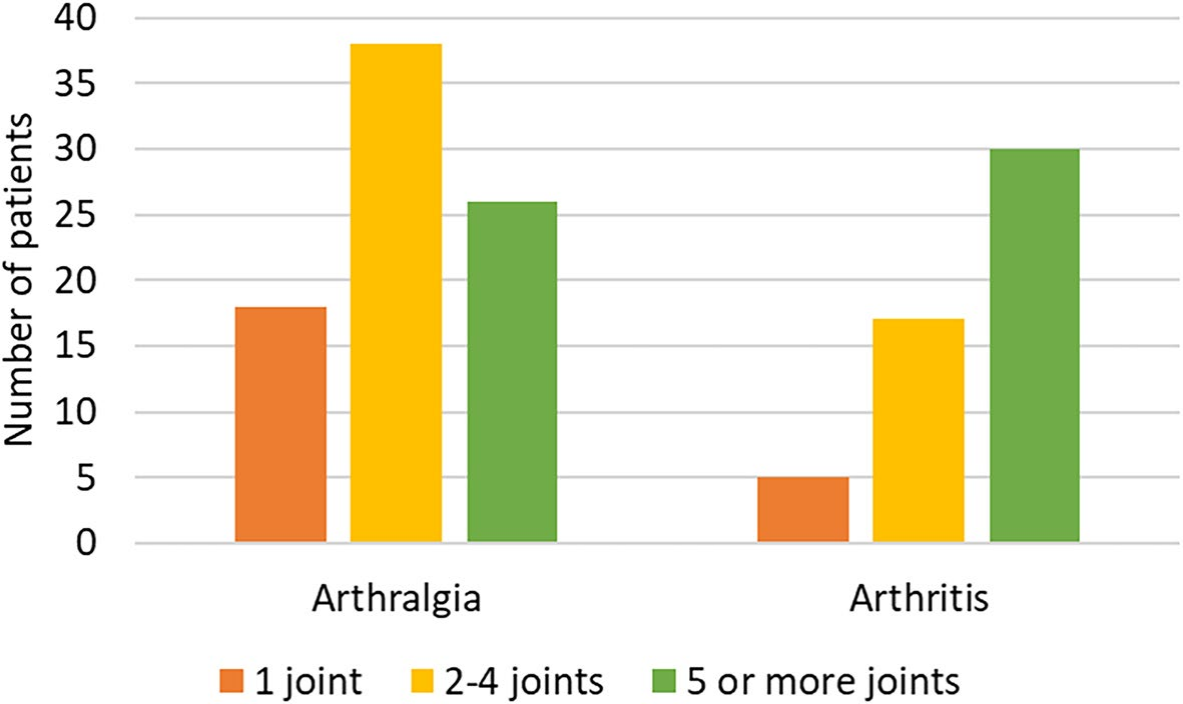
Distribution of joint involvement in childhood-onset systemic lupus erythematosus patients experiencing joint pain (*n* = 134)

Arthralgia was noted in 82 (61.2%) patients, which mainly exhibited oligoarthralgia, primarily affecting large joints such as the knees, wrists, and ankles. Arthritis was diagnosed in 52 (38.8%) patients, with a majority experiencing polyarthritis, primarily involving the knees and fingers. Fig. 1 illustrates the distribution of joint involvement among patients with SLE who experienced joint pain.

For MSK complications, avascular necrosis was confirmed in 16 (4.9%) patients, predominantly involving the bilateral hips. Septic arthritis was identified in 4 (1.2%) patients and was caused by Group D *Salmonella* spp. (2 patients), *Neisseria gonorrhoeae* (1 patient), and methicillin-resistant *Staphylococcus aureus* (1 patient). Concurrent osteomyelitis was detected in a patient with *Salmonella*-induced septic arthritis. Low bone mass was identified in 12 out of 33 individuals, as assessed by dual-energy X-ray absorptiometry. Table 3 summarizes the MSK complications observed in c-SLE patients. Additionally, subgroup analyses to compare between patients with and without septic arthritis, osteomyelitis or avascular necrosis were performed. There were no significant differences in the duration from onset of SLE symptoms to SLE diagnosis between the patients with and without septic arthritis, osteomyelitis or avascular necrosis; median (IQR) 1.0 (0.9–1.0) month vs 1.0 (0.7–1.9) month, p = 0.556. Patients with septic arthritis, osteomyelitis or avascular necrosis had significantly longer treatment duration than patients without these complications; median (IQR) 22.3 (20.1–28.6) months vs 11.3 (5.6–18.1) months, *p* < 0.001.

**Table 3:** Profile of musculoskeletal complications throughout the disease course among childhood-onset systemic lupus erythematosus patients (*N* = 321)

Musculoskeletal complications	n (%)
Any complications	31 (9.6)
Avascular necrosis	16 (4.9)
Bilateral hips	9
Unilateral hip	5
Knee	1
Talus	1
Low bone mass*	12 (3.7)
Septic arthritis	4 (1.2)
Group D *Salmonella* spp.	2
* Neisseria gonorrhoeae*	1
Methicillin-resistant *Staphylococcus aureus*	1
Osteomyelitis	1 (0.3)

^*^Assessed by dual-energy X-ray absorptiometry in 33 patients

## Discussion

Our investigation into the MSK manifestations and their patterns in 321 patients with c-SLE revealed that nearly half of the children and adolescents with SLE exhibited MSK manifestations at the initial diagnosis. The predominant MSK symptom was joint pain, with a higher incidence of arthralgia than arthritis. The symmetrical involvement of both large and small joints was a notable characteristic. Furthermore, patients who presented with MSK manifestations were typically older at the time of SLE diagnosis, more likely to exhibit non-scarring alopecia, neuropsychiatric involvement, and had higher SLE disease activity at diagnosis.

MSK complaints are frequently reported by SLE patients [2, 3, 19]. Joint pain is a particularly distressing symptom and is linked to a diminished quality of life [3, 5]. In our cohort, 41.7% of patients with c-SLE had MSK manifestations at the time of their SLE diagnosis. The prevalence of MSK manifestations in c-SLE patients has been shown to vary widely across countries: 28.5% in China [20], 30.5% in the United Kingdom [21], 41.8% in the United States [22], 56.3% in Singapore [23], 61.4% in Egypt [24], 56.6–70.4% in Turkey [25, 26] and 71% in India [27]. This variation in prevalence rates can be attributed to differences in ethnic backgrounds and the criteria used to define MSK manifestations across studies.

In adult SLE patients, lupus arthropathy has been categorized into three phenotypes: non-deforming non-erosive arthritis, deforming arthritis (Jaccoud’s arthropathy), and rheumatoid-like erosive arthritis (Rhupus syndrome) [2]. Intriguingly, our decade-long study revealed the rarity of Jaccoud’s arthropathy and Rhupus syndrome in patients with c-SLE, with no instances of these phenotypes observed. Furthermore, we noted a higher prevalence of arthralgia than of arthritis among c-SLE patients experiencing joint pain, a finding that contrasts with adult SLE studies. For instance, Ceccarelli et al. reported that in adult SLE patients, 26.8% experienced arthralgia, while 73.2% presented with arthritis [28]. Among our cohort with arthritis, polyarthritis affecting the knees and fingers was most prevalent and was observed in 57.7% of patients. This finding aligns with that of Sakamoto et al., who identified polyarthritis as the most frequent presentation in 59% of c-SLE patients [7]. Sener et al. described asymmetric polyarticular erosive arthritis associated with Rhupus syndrome in patients with c-SLE, although 60% of their study population had coexisting juvenile idiopathic arthritis [29]. Our study showed that muscle involvement was less common than joint involvement, revealing myalgia and myositis in only 6% and 2.2% of the c-SLE patients, aligning with findings by Sahin et al., which reported myositis in 5.4% [26]. Additionally, our findings indicated that tenosynovitis and enthesitis were uncommon in patients with c-SLE. These observations suggest that lupus arthropathy in patients with c-SLE is different from that in adults, potentially necessitating unique classification criteria.

Our patients with c-SLE exhibiting MSK manifestations were generally older at SLE diagnosis and had higher prevalence rates of non-scarring alopecia, neuropsychiatric involvement, and elevated SLE disease activity at the initial diagnosis. One study revealed a correlation between erosive arthritis and a cluster of neuropsychiatric symptoms, renal involvement, and serositis [28]. Additionally, Nelson et al.’s study compared early-onset SLE (before age 10) with peri-adolescent onset SLE (age 10 and above), revealing that arthritis was more prevalent in the peri-adolescent group (42.9%) than the early-onset SLE group (37%) [22]. This suggests that the prevalence of arthritis increases with age, a trend that is consistent with our findings. Sakamoto et al. observed that c-SLE patients initially diagnosed with chronic arthritis exhibited greater MSK damage at their final assessment than did those without chronic arthritis [7]. This finding suggests a correlation between extended disease duration and increased MSK damage, especially in patients with chronic arthritis from the onset of the disease. The association of MSK manifestations in c-SLE patients with non-scarring alopecia and neuropsychiatric involvement in our study points to distinct patterns of organ involvement in c-SLE patients.

In our study, MSK complications observed during the disease course included avascular necrosis, low bone mass, and septic arthritis/osteomyelitis. Avascular necrosis was identified in 4.9% of patients, predominantly affecting the hips, corroborating the findings of a study by Yang et al. [30]. Nonetheless, our reported prevalence was lower than the 10.2% reported by Sit et al. [9], which could be attributed to the longer duration of SLE disease in their cohort (median of 7.8 years) than in our cohort (median of 4.4 years). The variation might also be due to differences in exposure to systemic corticosteroids, a known risk factor for avascular necrosis and other forms of disease damage [9]. The longer duration of treatment could be possibly be another factor associated with these MSK complications, as shown from our study. Additionally, the reduced incidence of avascular necrosis in our study may reflect underdetection in asymptomatic patients. Clinicians must remain vigilant for avascular necrosis, given its potential to inflict significant damage and disability in c-SLE patients.

Our findings also highlighted bone and joint infections as significant MSK complications in patients with c-SLE, with Group D *Salmonella* spp. identified as the most prevalent pathogen in half of our patients with septic arthritis. This is in line with the findings of Huang et al., who reported similar rates of *Salmonella* and non-*Salmonella* pathogens [31]. Qiao et al. also noted that *Salmonella* spp., *Staphylococcus aureus*, and *Mycobacterium* spp. are common causative agents of septic arthritis in SLE patients [32]. Interestingly, despite Thailand being a tuberculosis-endemic area, we did not identify any cases of tuberculosis arthritis in our c-SLE cohort. This may be due to routine screening for tuberculosis and latent tuberculosis infection before initiating corticosteroids or immunosuppressive treatments in our practice.

The limitations of our study warrant consideration. The retrospective nature of our 10-year cohort study introduces potential challenges, including missing data, information bias, and possible confounders that could affect the accuracy of our findings. Additionally, as this was conducted at a single center, our study population may not fully represent the broader demographic population. However, our study was conducted at Thailand’s largest university-based tertiary hospital, which receives referrals nationwide. Another limitation is that not utilizing advanced imaging techniques, such as ultrasonography or magnetic resonance imaging, to detect subclinical inflammation may have resulted in an underestimation of subtle joint inflammation or erosions [33]. Furthermore, MSK complications such as asymptomatic avascular necrosis, may have been underreported, particularly in patients without MSK complaints. Similarly, low bone mass might not have been fully captured, given that not all c-SLE patients underwent routine dual-energy X-ray absorptiometry scans. Additionally, the data regarding treatment medications were not included in this study. Therefore, further studies to identify the treatment factors associated with MSK complications should be performed. Despite these limitations, our study offers valuable insights into MSK phenotypes specific to c-SLE within the real-world context of a Southeast Asian country.

## Conclusions

In our study, nearly half of the children and adolescents with c-SLE exhibited MSK manifestations at the initial diagnosis. The symmetrical involvement of both large and small joints was common, whereas Rhupus syndrome and Jaccoud’s arthropathy were infrequent. There is a significant need for long-term monitoring of MSK complications, particularly avascular necrosis. Healthcare professionals managing c-SLE patients must be vigilant about MSK manifestations from the outset and continuously monitor for MSK complications throughout disease progression, as these complications can lead to considerable patient disability and damage. The adoption of advanced imaging techniques, such as ultrasound and magnetic resonance imaging, could prove invaluable in detecting subtle inflammation, early joint erosion, and the early onset of avascular necrosis in c-SLE patients. The criteria for defining lupus arthropathy in patients with c-SLE require further refinement and development.

## Data Availability

Data are available from the corresponding author upon reasonable request.

## Abbreviations

c-SLE: Childhood-onset systemic lupus erythematosus
BMD: Bone mineral density
MSK: Musculoskeletal
SLE: Systemic lupus erythematosus
SLEDAI-2K: Systemic Lupus Erythematosus Disease Activity Index 2000
